# Minutiae Matching with Privacy Protection Based on the Combination of Garbled Circuit and Homomorphic Encryption

**DOI:** 10.1155/2014/525387

**Published:** 2014-02-24

**Authors:** Mengxing Li, Quan Feng, Jian Zhao, Mei Yang, Lijun Kang, Lili Wu

**Affiliations:** ^1^School of Communication and Electronic Engineering, Hunan City University, Yiyang, Hunan 41300, China; ^2^Engineering College, Gansu Agricultural University, Anning District, Lanzhou 730070, China; ^3^College of Information Sciences and Technology, Gansu Agricultural University, Anning District, Lanzhou 730070, China

## Abstract

Biometrics plays an important role in authentication applications since they are strongly linked to holders. With an increasing growth of e-commerce and e-government, one can expect that biometric-based authentication systems are possibly deployed over the open networks in the near future. However, due to its openness, the Internet poses a great challenge to the security and privacy of biometric authentication. Biometric data cannot be revoked, so it is of paramount importance that biometric data should be handled in a secure way. In this paper we present a scheme achieving privacy-preserving fingerprint authentication between two parties, in which fingerprint minutiae matching algorithm is completed in the encrypted domain. To improve the efficiency, we exploit homomorphic encryption as well as garbled circuits to design the protocol. Our goal is to provide protection for the security of template in storage and data privacy of two parties in transaction. The experimental results show that the proposed authentication protocol runs efficiently. Therefore, the protocol can run over open networks and help to alleviate the concerns on security and privacy of biometric applications over the open networks.

## 1. Introduction

Biometric characteristic, such as fingerprint, face, and iris, has been used to a higher level of security in order to cope with an increasing demand for reliable and highly usable information security systems. Currently, most practical biometric systems handle biometric data locally, or in secure local network. When they are migrated directly to an open network which is in partly secure or insecure environments, the inherent risks of privacy and security of traditional biometric technologies will be blown up. Jain et al. [1] analyze the vulnerabilities about biometrics to intrinsic failures and potential attacks by adversaries. One of the most serious risks is compromising the template database, which will exert a disastrous impact on the whole authentication system. Besides, a new challenge of privacy and security arises since a remote server and a user may not trust each other before the authentication. Their respective data should not be directly delivered to each other as in a usual biometric system. These data, as the private data, must not be leaked to each other during the authentication. Therefore, the template and the privacy protection are the key challenges for any biometric system deployed over the open network and must be properly addressed.

Among many solutions to privacy-preserving biometrics, some schemes focus on constructing a transformed template with noninvertible transformation, including fuzzy commitment [2], fuzzy vaults [3], biohashing [4], fuzzy sketch/fuzzy extractor [5], cancelable template [6], and random local region descriptor (RLRD) [7]. In these schemes, the researchers try to prevent biometric information being revealed from the transformed template by one-way transformation. However, some of them, for example, fuzzy vault, biohashing, and cancelable template, are proven to be vulnerable to attacks [8–10]. Though fuzzy sketch scheme is theoretically complete and secure, it complies with stronger requirements than what suffices in practice, which leads to degradation of accuracy.

Some researchers employ cryptographic technique to achieve privacy protection, which has a solid theoretical foundation. They have designed privacy-preserving protocols by resorting to secure two-party computation, where two nontrusted parties cooperate to carry out a computation without revealing their own inputs. Bringer and Chabanne [11] proposed a biometric authentication protocol which can protect the sensitive relationship between the biometric template and the relevant pseudorandom username, which is based on the homomorphic properties of Goldwasser-Micali and Paillier cryptosystems. Tang et al. [12] developed two concepts of biometric privacy called identity privacy and transaction anonymity, respectively, and presented an authentication protocol employing Private Information Retrieval protocols which was based on the ElGamal cryptosystems. In both works, however, the type of biometrics is limited to those which can be represented as binary strings because their protocols only computed the Hamming distance between biometric templates in the encryption domain. Erkin et al. [13] designed a privacy-preserving face recognition protocol based on additive homomorphic encryption, in which Euclidean distances between feature vectors or finding a minimum are computed by using the homomorphic property of the cipher texts. The protocol of Erkin requires large network traffic and a large memory, which makes those systems less practical. Sadeghi et al. [14] improved the performance by combining homomorphic encryption with garbled circuits. Barni et al. [15] employed fingercode [16] as a feature along with the technique similar to Sadeghi et al. [14] to realize privacy-preserving system for fingerprint-based identification. Huang et al. [17] provided further improvement for the privacy-preserving identification protocol by elaborate optimizations such as packing method, carefully integrating the subtraction and comparison computations, and backtracking technique. Upmanyu et al. [18] designed “blind” authentication protocol in which some classifiers such as linear, support vector machines, and neural networks were realized by using multiplicative homomorphic encryption. However, the experience of biometric recognition proved that, in most cases, direct matching shows a better performance than classifiers because, in practical applications, it is difficult to gather enough biometric samples of one person to train the classifier.

In this paper, we propose a solution to fingerprint authentication with providing protection for both template and privacy. In particular, we focus on the realization of fingerprint authentication in a secure framework by implementing minutiae matching instead of fixed-length features as in the existed schemes [16–18]. We address the problem of minutiae matching in the encrypted domain by combining homomorphic encryption with gabled circuit. In the proposed scheme, the template of a user, stored on the server, is encrypted by the user's private key, even the server learning nothing about the genuine information of the user. Therefore, our scheme shows a better performance in privacy protection than the existed schemes [16, 17].

## 2. Preliminaries

The primary cryptographic tools we use are homomorphic encryption, oblivious transfer, and garbled circuits. We briefly summarize each of these standard techniques here.

### 2.1. Additively Homomorphic Encryption

Let [*x*] denote encryption of *x* with a public key. Our constructions use a semantically secure public-key encryption scheme that preserves the group homomorphism of addition and allows multiplication by a constant. This property, which is obtained by the additively homomorphic encryption schemes, supports the following operations that can be performed without knowledge of the private key: (1) Given the encryptions [*a*] and [*b*], we can efficiently compute [*a* + *b*] = [*a*][*b*]. (2) Given that a constant *c* belongs to the same group, we can compute [*c* · *a*] = [*a*]^*c*^.

There are many public-key cryptosystems satisfying the above properties. In our implementation, we use Paillier's cryptosystem [19] which has plaintext space *Z*_*N*_ and cipher text space *Z*_*N*^2^_*, where *N* is a *T*-bit RSA modulus and *T* is the bits length of RSA.

### 2.2. Oblivious Transfer

1-out-of-2 oblivious transfer (*OT*_1_^2^) allows a sender, holding strings *s* ^0^, *s* ^1^, to transfer to a receiver, holding a selection bit *b*, exactly one of the inputs *s* ^*b*^. The receiver learns nothing about *s* ^1−*b*^, and the sender has no idea of *b*. Parallel *OT*_1_^2^ of *m* *l*-bit strings is denoted as *OT*_*l*_^*m*^. For *i* = 1,…, *m*, the sender inputs a pair of *l*-bit strings *s*_*i*_^0^, *s*_*i*_^1^ ∈ {0,1}^*l*^ and the receiver inputs *m* choice bits *b*_*i*_ ∈ {0,1}. At the end of the protocol, the receiver learns about the chosen string *s*_*i*_^*b*_*i*_^, but nothing about *s*_*i*_^1−*b*_*i*_^ whereas the sender learns nothing about the choice *b*_*i*_. Oblivious transfer has been studied extensively. In this scheme, we use oblivious transfer extension scheme of Ishai et al. [20] which serves to efficiently reduce the number of computationally expensive public-key operations of *OT*_*l*_^*m*^ to be independent of *m*.

### 2.3. Garbled Circuits

Garbled circuit [21, 22] allows two parties holding inputs *x* and *y*, respectively, to evaluate an arbitrary function *f*(*x*, *y*) without leaking any information about their inputs. The basic idea is that a server creates an “encrypted” version of the circuit *C* to compute *f*, and then a client obliviously computes the output of the circuit. In more detail, for each wire *w*_*i*_ of *C*, the server randomly chooses two secrets, ${\tilde{w}}_{i}^{0}$ and ${\tilde{w}}_{i}^{1}$, where ${\tilde{w}}_{i}^{j}$ is called garbled value of *w*_*i*_'s value*j*. Further, for each gate *G*_*i*_ of *C*, the server creates a garbled table ${\tilde{T}}_{i}$ which records a collection of the garbled values corresponding to the output wires of *G*_*i*_ with those corresponding to the input wires. All ${\tilde{T}}_{i}$s are transferred to a client as well as the garbled values of the server' input. The client gets the garbled values corresponding to his input by *OT* protocol from the server. Then, the client can evaluate the garbled circuit to obtain the garbled output simply gate by gate, using the garbled tables ${\tilde{T}}_{i}$s.

Some optimizations can be applied to the standard garbled circuit protocol. A powerful technique is “free XOR” scheme [23, 24] which eliminates the need to garble XOR gates, so XOR gates become “free,” incurring no communication or cryptographic operations. Another efficient approach [25] can reduce the size of a garbled table from four to three cipher texts for a 2-input-and-1-output gate, thus saving 25% of network bandwidth.

## 3. Overview

In the existing schemes [13–15, 17], the authors only considered protecting the privacy of biometric data of a user and a server, without taking the template protection into consideration. Although biometrics is assumed as public data, it should not be easy to obtain the biometric data by compromising a central server. Fingerprint is one of the biometric characteristics with the highest level of reliability. Barni et al. [15] and Huang et al. [17] both take fingercode as the feature, whose length is fixed. This helps to reduce the computational and communicational complexity. In fact, the minutiae set is the most popular feature used in practical systems because minutiae-based matching is more robust to distortions frequently encountered in practical applications, so these systems usually achieve a good accuracy. Minutiae are the endpoints and bifurcations of fingerprint ridges. Each minutia can be represented as (*x*, *y*, *θ*) triplet, where (*x*, *y*) is the location of the minutia and *θ* angle of the associated ridge (0 ≤ *θ* < 360°). A template of minutiae is represented as a set of points in the three-dimensional. Fingerprint matching can be reduced to finding the paired points problem. Let *M* ^*T*^ = {(*x*_*i*_, *y*_*i*_, *θ*_*i*_) | 1 ≤ *i* ≤ *N*_*T*_} and *M* ^*Q*^ = {(*x*_*j*_′, *y*_*j*_′, *θ*_*j*_′)1 ≤ *i* ≤ *N*_*Q*_} denote the template and query, respectively. A minutiae-based fingerprint matching algorithm usually returns the number of matched minutiae on both *M*_*T*_ and *M*_*Q*_ to generate similarity scores. In this paper, the matching score *S*_*M*_ is calculated as follows:

(1) $$\begin{array}{c} S_{M}=\frac{100\times N_{M}}{\max\left(N_{T},N_{Q}\right)}, \end{array}$$

where *N*_*M*_ is the number of paired minutiae. If *S*_*M*_ is greater than or equal to a predefined threshold *T*_*M*_; then the query and the template can be considered coming from the same finger.

A minutia *M*_*i*_ = (*x*_*i*_, *y*_*i*_, *θ*_*i*_) in *M* ^*T*^ and a minutia *M*_*j*_′ = (*x*_*j*_′, *y*_*j*_′, *θ*_*j*_′) in *M* ^*Q*^ are considered matching if the following conditions are satisfied:

(2) $$\begin{array}{c} d\left(\left(x_{i},y_{i}\right),\left(x_{j}^{\prime },y_{j}^{\prime }\right)\right)<T_{D}, \end{array}$$

(3) $$\begin{array}{c} \min\left(\left|\theta_{i}-\theta_{j}^{\prime }\right|,360-\left|\theta_{i}-\theta_{j}^{\prime }\right|\right)<T_{\theta }, \end{array}$$

where *d*( ) is a distance function and *T*_*D*_ and *T*_*θ*_ are the given thresholds. In this paper, we consider two distance metrics: (i) square of Euclidean distance *d*_*E*_((*x*_*i*_, *y*_*i*_), (*x*_*j*_′, *y*_*j*_′)) = (*x*_*i*_ − *x*_*j*_′)^2^ + (*y*_*i*_ − *y*_*j*_′)^2^ (for simplicity, we still call Euclidean distance) and (ii) city block distance *d*_*B*_((*x*_*i*_, *y*_*i*_), (*x*_*j*_′, *y*_*j*_′)) = |*x*_*i*_ − *x*_*j*_′| + |*y*_*i*_ − *y*_*j*_′|. However, for a given minutia belonging to *M* ^*Q*^, the above approach might find at least one matching result belonging to *M* ^*T*^, but, in fact, at most one is correct. In this paper, for a minutia *M*_*j*_′ ∈ *M* ^*Q*^ and those minutiae belonging to *M* ^*T*^ satisfying (2) and (3), we choose the closest to *M*_*j*_′ as the matched minutia.

We design the privacy-preserving protocol based on the aforementioned matching algorithm, which works in the two-party setting in the semihonest attacker model. In this model, the participants do not deviate from their protocol but may use any information they obtain to their own advantage. Suppose that Bob (the server) holds a database containing the template of the users. To protect the user's privacy, however, the template is not the original feature but its encrypted version. Thus, even Bob does not learn the user's biometric information. Alice (the user) can update her template by choosing a new private key. When Bob receives Alice's request for authentication, he first retrieves the encrypted template from his database. During the interactive authentication protocol, Alice provides the fresh minutiae *M* ^*Q*^ as the inputs. She trusts Bob to correctly perform the matching algorithm but is unwilling to expose her information of fingerprint to Bob. The protocol consists of two phases: the first one is related to the blind distance computation, which is carried out by homomorphic encryption (Section 4); the second one is related to minutiae matching, which is implemented by garbled circuit (Section 5). At the end of protocol, Alice obtains the number of the matched minutiae and returns it to Bob. However, the number is represented as the garbled value, so Alice knows nothing about the genuine value. Bob decrypts it and computes the matching score. For the sake of simplicity, we just describe the content concerning distance computation and minutiae matching.

## 4. Blind Distance Computation 

In this section, we present two protocols which compute the two kinds of blind distances of Euclidean and city block, respectively, which is the first phase in our authentication process.

### 4.1. Euclidean-Distance Protocol

*Basic.* Alice computes the distance between each minutia in *M* ^*Q*^ and that in *M* ^*T*^ with the help of Bob. As mentioned in (2) and (3), there are two distances needing to be calculated: the spatial distance and the directional difference. The spatial distance discussed in this section is Euclidean distance. *M* ^*T*^ is the encrypted version with Alice's private key while *M* ^*Q*^ is in the clear. Here we denote the encrypted template as *EM* ^*T*^ = {*EM*_*i*_ = ([*x*_*i*_], [*x*_*i*_^2^], [*y*_*i*_], [*y*_*i*_^2^], [*θ*_*i*_]) | 1 ≤ *i* ≤ *N*_*T*_}. *EM* ^*T*^ is held by Bob. Since Alice holds the private key, to keep his privacy, Bob can blind *M* ^*T*^ in advance and Alice can only compute the blind distance. To do so, he blinds *EM* with the uniformly random numbers *r*_1_, *r*_2_, *r*_3_, *r*_4_ from the plaintext space to get the following cipher texts by using the homomorphic property: [*a*] = [*x*][*r*_1_] = [*x* + *r*_1_], [*b*] = [*y*][*r*_2_] = [*y* + *r*_2_], [*c*] = [*x* ^2^][*r*_3_] = [*x* ^2^ + *r*_3_], [*d*] = [*y* ^2^][*r*_4_] = [*y* ^2^ + *r*_4_]. Then he sends these cipher texts to Alice. Alice decrypts them to get *a*, *b*, *c*,and *d*. She calculates

(4) ed=c+d−2x′a−2y′b+x′2+y′2=x2−2xx′+x′2+y2−2yy′+y′2+r3+r4−2r1x′−2r2y′.

To further compute the blind distance, Alice needs 2*r*_1_ *x*′ + 2*r*_2_ *y*′ which can be easily gotten by interacting with Bob: Alice transfers [2*x*′] and [2*y*′] to Bob. The latter computes and returns [*t*] = [2*x*′]^*r*_1_^[2*y*′]^*r*_2_^[*r*_5_], where *r*_5_ ∈_*R*_ *Z*_*N*_. It is easy to observe that [*t*] = [2*x*′*r*_1_ + 2*y*′*r*_2_ + *r*_5_]. Alice decrypts [*t*] and calculates the blind distance *bd* = *ed* + *t* = *d*_*E*_ + *r*_3_ + *r*_4_ + *r*_5_ = *d*_*E*_ + *r*.

Bob further blinds [*θ*] with a random number by computing *e* = [*r*_6_][*θ*] = [*r*_6_ + *θ*], where *r*_6_ is a random number and then transfers it to Alice.

*Improvement.* In order to improve the efficiency of the above approach, we choose the shorter random masks and pack multiple values into a single cipher text. Assume that *x*(*x*′), *y*(*y*′), *x* ^2^, *y* ^2^, and *θ* are *ρ*-, *ρ*-, 2*ρ*-, 2*ρ*-, and *μ*-bit positive integers, respectively, and the random masks *r*_1_, *r*_2_, *r*_3_, *r*_4_, *r*_5_, and *r*_6_ are *η*_*r*_1__-, *η*_*r*_2__-, *η*_*r*_3__-, *η*_*r*_4__-, *η*_*r*_5__-, and *η*_*r*_6__-bit positive integers, respectively. The resulting blind values *x* + *r*_1_, *y* + *r*_2_, *x* ^2^ + *r*_3_, *y* ^2^ + *r*_4_, and *θ* + *r*_6_ can be packed into a single cipher text. The cross item such as *t* = 2*x*′*r*_1_ + 2*y*′*r*_2_ + *r*_5_ can also be packed. The storage size of the encrypted minutiae template can also be reduced by using the packing technique. That is, when generating the encrypted template *EM* ^*T*^, we firstly pad some zeros before *x*, *y*, *x* ^2^, *y* ^2^, and *θ* to increase their lengths to *η*_*r*_1__ + 1, *η*_*r*_2__ + 1, *η*_*r*_3__ + 1, *η*_*r*_4__ + 1, *η*_*r*_6__ + 1, respectively, and concatenate them together. As described later, we have *η*_*r*_1__ = *ρ* + *δ*, *η*_*r*_2__ = *ρ* + *δ*, *η*_*r*_3__ = 2*ρ* + *δ*, *η*_*r*_4__ = 2*ρ* + *δ*, *η*_*r*_6__ = *η* + *δ* (*δ* is a security parameter which will be explained later). Then a unit chunk representing a minutia *M*_*i*_ = (*x*_*i*_, *y*_*i*_, *θ*_*i*_) is written down as: *mp*_*i*_ = 0^*δ*+1^||*x*_*i*_||  0^*δ*+1^||*y*_*i*_||0^*δ*+1^||*x*_*i*_^2^||0^*δ*+1^||*y*_*i*_^2^||0^*δ*+1^||*θ*_*i*_. The purpose of padding *δ* + 1 zeroes before each component is to prevent the possible overflow when the component is added to the corresponding mask. Therefore, one cipher text can contain *N*_*P*_ = ⌈*T*/(6*ρ* + *μ* + 5*δ* + 5)⌉ minutiae. The number of cipher texts of *EM* ^*T*^ is only *N*_*M*_ = ⌊*N*_*T*_/*N*_*P*_⌋. Compared with the basic approach, the method saves (1 − *N*_*M*_/5*N*_*T*_)% storage space. We rewrite *EM* ^*T*^as: *EM* ^*T*^ = {*cp*_1_ = [*mp*_1_||⋯|| *mp*_*N*_*P*__],…, *cp*_*N*_*M*__ = [*mp*_(*N*_*M*_−1)*N*_*P*_+1_||⋯|| *mp*_*N*_*T*__]}. These methods reduce the communicational and computational complexity because each cipher text carries multiple blind minutiae.

*Enrollment.* When Alice registers herself to Bob, she creates an encrypted template *EM* ^*T*^ by employing the aforementioned method. She sends *EM* ^*T*^ to Bob who stores *EM* ^*T*^ in a safe database.

*Protocol.* In the authentication phase, Alice and Bob carry out the protocol Euclidean-Distance to compute the blind distances of the minutiae (Algorithm 1). The detail is given as follows. For simplicity, we assume that two parties have learned the number of minutiae in the template and the query, that is, *N*_*T*_ and *N*_*Q*_, and exchanged the public key.

*Packing Size.* We have supposed that *x*, *y*, *x* ^2^, *y* ^2^, and *θ* are *ρ*, *ρ*, 2*ρ*, 2*ρ*, and *μ* bits, respectively, and random masks *r*_1_, *r*_2_, *r*_3_, *r*_4_, *r*_6_ are *η*_*r*_1__, *η*_*r*_2__, *η*_*r*_3__, *η*_*r*_4__, and *η*_*r*_6__ bits, respectively. To keep statistical security, we need *η*_*r*_1__ > *ρ*, *η*_*r*_2__ > *ρ*, *η*_*r*_3__ > 2*ρ*, *η*_*r*_4__ > 2*ρ*, and *η*_*r*_6__ > *μ*. In fact, our random masks are longer than the corresponding blind values by *δ* bits; that is, *η*_*r*_1__ = *ρ* + *δ*, *η*_*r*_2__ = *ρ* + *δ*, *η*_*r*_3__ = 2*ρ* + *δ*, *η*_*r*_4__ = 2*ρ* + *δ*, and *η*_*r*_6__ = *μ* + *δ*. Besides, we need to handle the possible overflow of the intermediate values in computation. Therefore, the length of these values will be determined as follows: *a*, *b*, *c*, *d*, and *e* (step 1) are *ρ* + *δ* + 1-, *ρ* + *δ* + 1-, 2*ρ* + *δ* + 1-, 2*ρ* + *δ* + 1-, and *μ* + *δ* + 1-bit values. The main task of the protocol is computing the cross item *t*_*i*,*j*_^4^ = 2*r*_*i*_^1^ *x*_*j*_′ + 2*r*_*i*_^2^ *y*_*j*_′ + *r*_*i*,*j*_^5^ (step 4 (i)). We need (*η*_*r*_1__ + *ρ* + 1) + 1 = 2*ρ* + *δ* + 2 bits to represent 2*r*_*i*_^1^ *x*_*j*_′ + 2*r*_*i*_^2^ *y*_*j*_′, and *η*_*r*_5__ = 2*ρ* + *δ* + 2 to represent the random mask *r*_*i*,*j*_^5^. Accordingly, it is clear that *λ* = *η*_*r*_5__ + 1 = 2*ρ* + *δ* + 3 bits are sufficient (step 2 (ii)). We pad *λ* − *ρ* − 1 zeros before 2*x*_*j*_′ and 2*y*_*j*_′ to form *λ*-bit *u*_*i*_^1^ and *u*_*i*_^2^ because this ensures that no overflow happens when computing 2*r*_*i*_^1^ *x*_*j*_′ + 2*r*_*i*_^2^ *y*_*j*_′ (step 2 (ii)). The blind squared-distance *bd*(*i*, *j*) and the blind orientation difference *bo*(*i*, *j*) are *λ*-bit and *μ* + *δ* + 1-bit, respectively.

*Correctness.* Here, we prove that the equality in Euclidean-Distance, *bd*(*i*, *j*) = *ed*_*ij*_ + *t*_*i*,*j*_^4^ = *d*_*E*_(*i*, *j*) + *r*_*ij*_ is established. In step 2 of the protocol, since *u*_*i*_^1^ is *λ* = 2*ρ* + *δ* + 3-bit, *r*_*i*_^1^, *ρ* + *δ*-bit, and 2*x*′, *ρ* + 1-bit binary string, respectively; in step 3,

(5) [B1]ri1=[u11||⋯||uNX1]ri1=[∑j=1NXuj1·2(NX−j)λ]ri1=[∑j=1NXuj1·ri1·2(NX−j)λ]=[∑j=1NX2xj′·ri1·2(NX−j)λ]=[2x1′ri1||2x2′ri1||⋯2xNX′ri1],[B2]ri1=[2xNX+1′ri1||⋯],

and so forth.

Similarly,

(6) $$\begin{array}{c} {\left[C_{1}\right]}^{r_{i}^{1}}={\left[u_{1}^{2}||\cdots ||u_{N_{X}}^{2}\right]}^{r_{i}^{1}}=\left[2y_{1}^{\prime }r_{i}^{2}||2y_{2}^{\prime }r_{i}^{2}||\cdots 2y_{N_{X}}^{\prime }r_{i}^{2}\right],\\ {\left[C_{2}\right]}^{r_{i}^{1}}=\left[2y_{N_{X}+1}^{\prime }r_{\text{i}}^{2}||\cdots \right], \end{array}$$

and so forth. So,

(7) [ti,11]=[B1]ri1[C1]ri2=[ri1B1+ri2C1]=[2x1′ri1+2y1′ri2||⋯||2xNX′ri1+2xNX′ri2],[ti,21]=[2xNX+1′ri1+2yNX+1′ri2||⋯],

and so forth.

One can further verify that

(8) [ti,13]=[ti,11+ti,12]=[2ri1x1′+2ri2y1′+ri,15||⋯||2ri1xNX′+2ri2yNX′  +ri,NX5],…,[ti,NB3]=[ti,NB1+ti,NB2]=[2ri1x(NB−1)NX+1′+2ri2y(NB−1)NX+1′+ri,(NB−1)NX+15||⋯||2ri1xNQ′+2ri2yNQ′+ri,NQ5].

Thus,

(9) ti,j4=2ri1xj′+2ri2yj′+ri,j5(step  4),bd(i,j)=edij+ti,j4=(xi−xj′)2+(yi−yj′)2+ri3+ri4+ri,j5=dE(i,j)+ri3+ri4+ri,j5=dE(i,j)+rij.

Obviously, *bo*(*i*, *j*) = *e*_*i*_ − *θ*_*j*_′ = *θ*_*i*_ − *θ*_*j*_′ + *r*_*i*_^6^, which is the blind difference between *θ*_*i*_ and *θ*_*j*_′.

*Security.* The security of the protocol depends on whether the short masks can adequately blind the values that Bob is unwilling to reveal to Alice. Since the addition in the homomorphic encryption is computed over the integers rather than modulo addition, we only obtain statistical hiding rather than perfect hiding. If *w* is *ρ*-bit integer and *r* is uniform *η*-bit integer, then *v* = *w* + *r* gives statistical security roughly 2^*ρ*−*η*^ for *w*, where *w* can stand for *x*, *y*, *x* ^2^, *y* ^2^, and *θ*. This probability can be lowered arbitrarily by choosing *η* properly. However, a longer *η* will increase computational and communicational complexity. Since *x*, *y*, and *θ* are *ρ*-, *ρ*-, and *μ*-bit integers, respectively, and the probability of guessing right *x*, *y*, and *θ* is 2^−*ρ*^, 2^−*ρ*^, and 2^−*μ*^, respectively, it is sufficient that *η*_*r*_1__ − *ρ*, *η*_*r*_2__ − *ρ* are not less than *ρ* and *η*_*r*_6__ − *μ* is not less than *μ*. Assume that *ρ* is equal to *μ*; thus, *δ* = *ρ* = *μ* is satisfied.

*Complexity.* Since the computational complexity of euclidean-distance is dominated by operations related to Paillier encryption, such as exponentiation with an exponent of length *T* (Exp), encryption (Enc), and decryption (Dec), we only take their costs into consideration. The overall complexity of Euclidean-Distance is given in Table 1. The computational complexity of the protocol can be further reduced. The operations in step 1 of the protocol can be precomputed, and, thus, Bob saves *N*_*M*_ Encryption.

### 4.2. City-Block-Distance Protocol

In this section, we present a protocol based on city block distance (Algorithm 2). As there are no quadric components in the computation of city block distance, the size of the encrypted template can also be reduced. Let us describe how to generate the encrypted template firstly. For a minutia *M*_*i*_ = (*x*_*i*_, *y*_*i*_, *θ*_*i*_), we pad *δ* + 1 zeroes before each component and concatenate them to *mp*_*i*_, where *mp*_*i*_ = 0^*δ*+1^||*x*_*i*_||0^*δ*+1^||*y*_*i*_||0^*δ*+1^||*θ*_*i*_. The encrypted template can be created as: *EM* ^*T*^ = {*cp*_1_ = [*mp*_1_||⋯||*mp*_*N*_*P*__],…, *cp*_*N*_*M*__ = [*mp*_(*N*_*M*_−1)*N*_*P*_+1_||⋯|| *mp*_*N*_*T*__]}, where *N*_*P*_ = ⌊*T*/(2*ρ* + *μ* + 3*δ* + 3)⌋ and *N*_*M*_ = ⌈*N*_*T*_/*N*_*P*_⌉. Compared with the template in Section 4.1, the size of this template is further reduced.

In the authentication phase, Alice and Bob firstly carry out the protocol City-Block-Distance to calculate a blind list for further computation of city block by using garbled circuits. We detail the protocol as follows.

Compared with Euclidean-Distance, this protocol is rather simple. It is easy to verify the correctness of the protocol by employing the homomorphic property. The secure analysis is similar to that of Euclidean-Distance.  Table 2 shows its complexity. The computational complexity can be further reduced by precomputing step 1 of the protocol.

## 5. Circuits for Minutiae Matching

Garbled circuit is employed to complete minutiae matching with respect to Section 3.  Section 5.1 describes the circuits related to Euclidean distance and Section 5.2 presents the circuits related to city block distance.

### 5.1. Circuits for Euclidean Distance

After the protocol Euclidean-Distance is carried out, Alice learns the blind Euclidean distance *bd*(*i*, *j*) = *d*_*E*_(*i*, *j*) + *r*_*ij*_ and the blind directional difference *bo*(*i*, *j*) = *θ*_*i*_ − *θ*_*j*_′ + *r*_*i*_^6^. The remaining tasks of authentication are implemented by using garbled circuits. The circuits firstly take off the random masks covered on *bd*(*i*, *j*) and *bo*(*i*, *j*) to get the Euclidean distances and the directional differences. Then, for each minutia (*x*_*i*_, *y*_*i*_, *θ*_*i*_) belonging to *M* ^*T*^(1 ≤ *i* ≤ *N*_*T*_), the closest matching minutia which belongs to *M* ^*Q*^ is found according to (2) and (3). To do so, the circuits choose the minutiae belonging to *M* ^*Q*^ as the candidates, whose directional differences are smaller than the threshold *T*_*θ*_ (see (3)). The candidates' Euclidean distances are then fed into the minimum circuit to find the minimum. And then the minimum is checked whether it is smaller than the threshold *T*_*D*_ (see (2)). Finally, the number of minutiae belonging to *M* ^*Q*^ meeting the above two conditions is counted. We adopt the efficient building blocks from [23, 24] to design our circuit: addition ADD, subtraction SUB, comparison CMP, and multiplexer MUX circuits.

**Figure 1:**
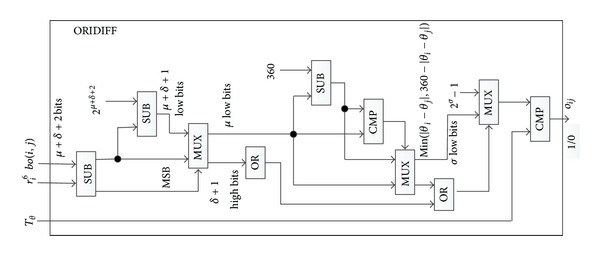
ORIDIFF circuit for computing (3).

Figure 1 shows the circuit ORIDIFF to take off the mask of *bo*(*i*, *j*) and compute the result according to (3). Alice's input is *bo*(*i*, *j*) and Bob's, *r*_*i*_^6^ and *T*_*θ*_. If *θ*_*i*_ − *θ*_*j*_′ is smaller than *T*_*θ*_, ORIDIFF outputs *oa*_*ij*_ = “1”; otherwise *oa*_*ij*_ = “0”. Since *bo*(*i*, *j*) is the result of *e*_*i*_ − *θ*_*j*_′, *bo*(*i*, *j*) is likely to be negative, and it must be represented as a signed integer. We represent it as a *μ* + *δ* + 2-bit integer in two's complement representation to cater to the requirement of circuit SUB [24], and so does *r*_*i*_^6^. In ORIDIFF, the first SUB(in Figure 1, from left to right) outputs the result of *θ*_*i*_ − *θ*_*j*_′. However, we want to get its absolute value according to (3). Since the result is represented in two's complement, if it is negative, the second SUB computes its magnitude by subtracting it from 2^*μ*+*δ*+2^. The most significant bit (MSB) of the output of the first SUB, which is the signed bit, controls the selection of the first MUX in ORIDIFF. If the MSB is “0,” the MUX chooses the output of the first SUB, otherwise the output of the second SUB. Thus, the first MUX outputs |*θ*_*i*_ − *θ*_*j*_′| which is smaller than 360°. However, the output of the MUX is *μ* + *δ* + 1 bits. Since the bits length of the orientation is *μ* bits, *μ* low bits of the output can be only preserved for the next computation. As *μ* + *δ* + 1 is significantly bigger than *μ*, this method substantially reduces the number of gates. The third SUB in ORIDIFF computes the result of 360 − |*θ*_*i*_ − *θ*_*j*_′|. And the second MUX in ORIDIFF outputs *od* = min⁡⁡(|*θ*_*i*_ − *θ*_*j*_′|, 360 − |*θ*_*i*_ − *θ*_*j*_′|). If there exists the forged input, the *δ* + 1 high bits of the output of the first MUX may not be zeros. When this happens, we need to set the output of ORIDIFF “0.” Besides, the bit lengths of *od* and *T*_*θ*_ are *μ* and *σ*, respectively, and *σ*is smaller than *μ*. So if the high order *μ* − *σ* bits are not zeros, *od* must be greater than *T*_*θ*_, and there is no need to compare the other bits. Considering the above two cases, in ORIDIFF, we compute the logical OR of high order *δ* + 2 bits of the output of the first MUX and high order *μ* − *σ* bits of the second MUX. If the result is 1, the third MUX chooses 2^*σ*−1^ as its output, otherwise, the low *σ* bits of the second MUX. The OR operation can be implemented by *λ* − *τ* − 1 two-inputs-OR gates one by one. At last, the output of the third MUX is compared against *T*_*θ*_, if it is greater than *T*_*θ*_, *o*_*ij*_; the output of ORIDIFF is set to 1, otherwise, 0, which will be further utilized to control the computation related to spatial distance. Bob generates *N*_*T*_ × *N*_*Q*_ ORIDIFF for Alice to evaluate (3) where *i* = 1,…, *N*_*T*_ and *j* = 1,…, *N*_*Q*_.

**Figure 2:**
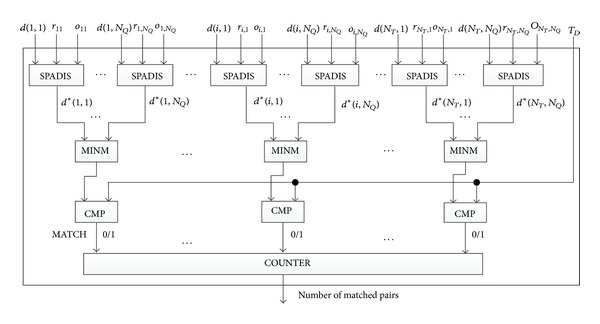
Finding matched minutiae and counting their number.

Figure 2 reveals the circuit of MATCH which serves to uncover the masks, find the matched minutiae, and count their number. The functions of the modules SPADIS, MINM, and COUNTER will be detailed later. Alice's inputs are blind squared-distances *bd*(1,1),…, *bd*(1, *N*_*Q*_),…, *bd*(*i*, 1),…, *bd*(*i*, *N*_*Q*_),…, *bd*(*N*_*T*_, 1),…, *d*(*N*_*T*_, *N*_*Q*_), and the corresponding *o*_11_,…, *o*_1,*N*_*Q*__,…, *o*_*i*,1_,…, *o*_*i*,*N*_*Q*__, *o*_*N*_*Q*_,1_,…, *o*_*N*_*T*_,*N*_*Q*__; Bob's inputs are random masks *r*_11_, …, *r*_1,*N*_*Q*__, …, *r*_*i*1_, …, *r*_*i*,*N*_*Q*__, …, *r*_*N*_*T*_,*N*_*Q*__, where *r*_*ij*_ = *r*_*i*_^3^ + *r*_*i*_^4^ + *r*_*i*,*j*_^5^ and 1 ≤ *i* ≤ *N*_*T*_,1 ≤ *j* ≤ *N*_*Q*_. Note that *bd*(*i*, *j*) is larger than *r*_*ij*_ and they are both *λ*-bit positive integers. However, the result of *bd*(*i*, *j*) − *r*_*ij*_ is the squared distance *d*_*E*_(*i*, *j*) which can be represented as 2**ρ**-bit integer (*λ* > 2*ρ*). Let *d*_*E*_(*i*, 1),…, *d*_*E*_(*i*, *N*_*Q*_)denote the distances between a minutia *M*_*i*_ in *M* ^*T*^ and each minutia in *M* ^*Q*^, respectively, (1 ≤ *i* ≤ *N*_*T*_). Among these distances, only the smallest one that simultaneously meets the requirement specified in (3) is picked out to compare with the threshold *T*_*D*_. Let *τ* denote its bits length (note that *τ* < 2*ρ*). To reduce the complexity of overall circuits, instead of directly comparing *d*_*E*_(*i*, *j*) with *T*_*D*_, we generate d*(*i*, *j*) which is only a *τ* bits value as follows and compare it with *T*_*D*_ later, which can avoid unnecessary bit operations:

(10)   d∗(i,j) ={2τ−1,if  bd(i,j)−rij≥2τ  or  oij=1τ low-order  bits  if  bd(i,j)−rij<2τ,oij=0,of  bd(i,j)−rij.

**Figure 3:**
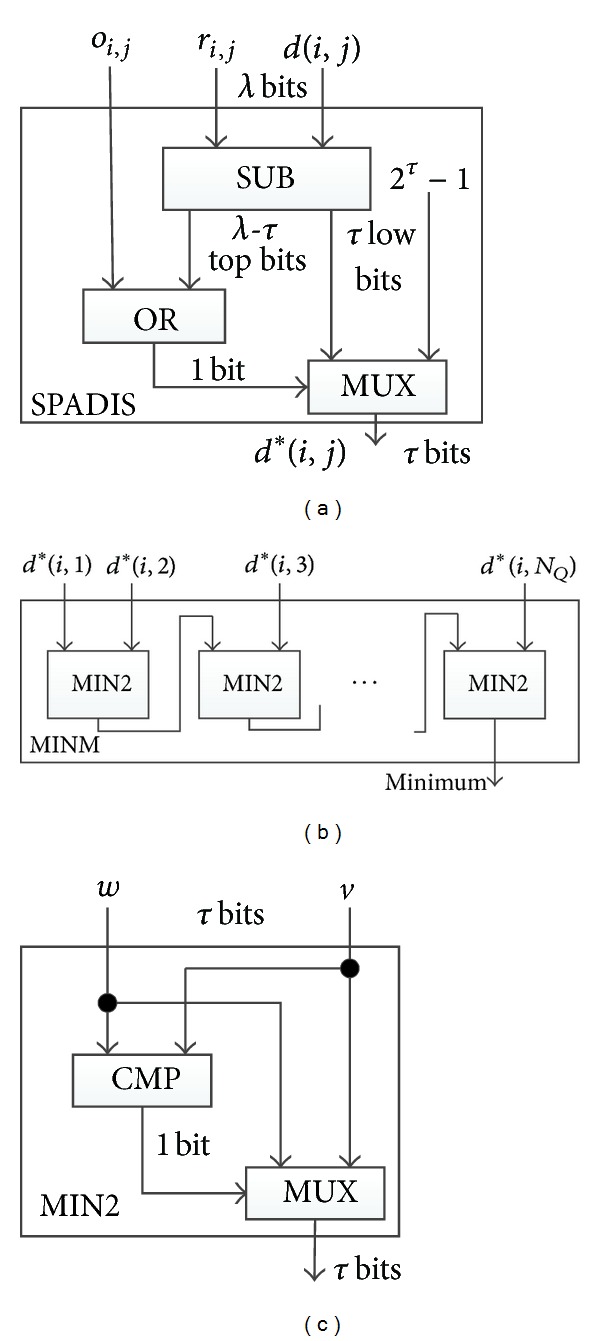
Structure of module SPADIS for Euclidean distance, MINM, and MIN2. (a) SPADIS, (b). MINM, and (c). MIN2.

The module SPADIS shown in Figure 3(a) uncovers the masks and compute d*(*i*, *j*). The logical OR of the high *λ* − *τ* bits of *bd*(*i*, *j*) − *r*_*ij*_ is computed. If the result is 1, *bd*(*i*, *j*) − *r*_*ij*_ must be greater than *T*_*D*_. Hence, there is no need to compare the other bits. The *λ* − *τ*-bit OR operation can be implemented by *λ* − *τ* − 1 two-inputs-OR gates one by one. Besides, if the bit *o*_*ij*_ is 1, it indicates that the orientation difference between *M*_*i*_ and *M*_*j*_ is too large to meet the requirement, so OR of *o*_*ij*_ with the result of the above OR operation is further computed. For simplicity, we draw only one OR block standing for these operations in Figure 3(a). The result of the OR block controls the MUX to select 2^*τ*^ − 1 or the low *τ* bits of *bd*(*i*, *j*) − *r*_*ij*_. Since *τ* is significantly smaller than *λ*, the method saves a mass of gates in MIN and CMP circuits.

The module of MINM is presented in Figure 3(b), which takes as the inputs d*(*i*, 1), …, d*(*i*, *N*_*Q*_) where 1 ≤ *i* ≤ *N*_*T*_ and picks out the minimum. The circuit MIN2 shown in Figure 3(c) is the functional unit of MINM, which compares two inputs *w* against *v* and selects the smaller one.

**Figure 4:**
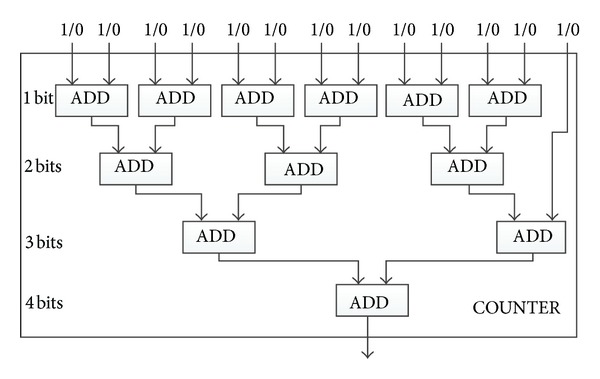
An example of COUNTER with *N*_*T*_ = 13.

In Figure 2, if the output of *i*th CMP is 1 (1 ≤ *i* ≤ *N*_*T*_), it indicates that there exists a minutia belonging to *M* ^*Q*^ that matches the *i*th minutia *M*_*i*_ belonging to *M* ^*T*^. The module of COUNTER further counts the number of these “1”s. Obviously, the number is at most *N*_*T*_, so *n* = ⌊log⁡*N*_*T*_⌋ bits are needed to represent it. To reduce the complexity, we do not use *N*_*T*_ *n*-bit adders one by one to construct COUNTER. Instead, we use a hierarchical structure, which includes *n* levels. The first level is composed of 1-bit ADDs with the number of ⌈*N*_*T*_/2⌉. The second level is composed of 2-bit ADD with the number of ⌈⌊*N*_*T*_/2⌋/2⌉, and so forth. The *n*th level is composed of only one *n*-bit ADD.  Figure 4 shows an example for constructing COUNTER with *N*_*T*_ being thirteen.

We can estimate the cost of circuits used in this section. The blocks here adopt the technique of “free” XOR [23, 24], which do not contribute significantly to the cost of garbled circuits since they need no communicational or cryptographic operations, so we just consider the number of non- XOR gates in the circuits.  Table 3 gives the number of non-XOR gates in each of the circuits and the total number.

To implement the authentication, Bob prepares a garbled version of the circuits described above and transfers them to Alice, as well as the garbled values of his inputs—the random masks and the thresholds, *T*_*θ*_ and *T*_*D*_. Alice carries out the OT protocol along with Bob to obtain the garbled values corresponding to her inputs—*bd*(*i*, *j*) and *bo*(*i*, *j*) for 1 ≤ *i* ≤ *N*_*T*_, 1 ≤ *k* ≤ *N*_*B*_. She first evaluates ORIDIFF and then MATCH. The final result is the garbled version of the number of the matched minutiae. She sends it to Bob who gets the actual value and computes the matching score according to (1). If the score is greater than the threshold *T*_*M*_, Bob will accept Alice's identity, otherwise, reject her.

### 5.2. Matching Circuit for City Block Distance

**Figure 5:**
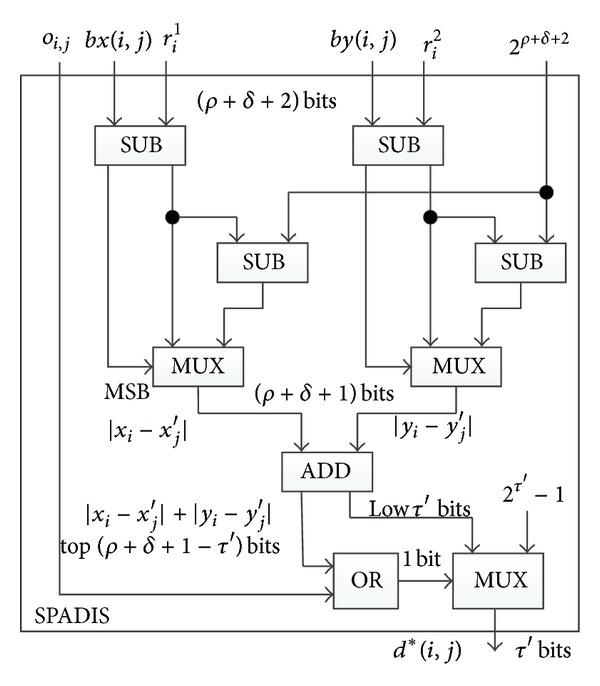
Structure of SPADIS for city block distance.

After the protocol City-Block-Distance is performed, Alice only get the intermediate data—the blind list {*bx*(*i*, *j*), *by*(*i*, *j*), *bo*(*i*, *j*) | 1 ≤ *i* ≤ *N*_*T*_, 1 ≤ *j* ≤ *N*_*Q*_}. In this section, we present the circuit to complete the computation of city block distance as well as the other subtasks. Firstly, Alice computes the circuits containing *N*_*T*_ × *N*_*Q*_ ORIDIFF to take off the random masks of *bo*(*i*, *j*) and compute the directional differences, select the minutiae belonging to *M* ^*Q*^, whose directional differences are smaller than *T*_*θ*_ as the candidates. Secondly, she uses the circuits shown in Figure 2 to calculate the distances, find the minima among the candidates, and check whether they are smaller than *T*_*D*_. However, the module SPADIS of Figure 2 should be replaced with Figure 5 designed for city block distance. Finally, she counts the number of the matched minutiae by using COUNTER described in Section 5.1.

SPADIS in Figure 5 takes as the inputs *bx*(*i*, *j*), *r*_1_^*i*^, *by*(*i*, *j*), *r*_2_^*i*^, and *o*_*i*,*j*_. It firstly takes off the random masks (*r*_1_^*i*^, *r*_2_^*i*^) on *bx*(*i*, *j*) and *by*(*i*, *j*). Note that *bx*(*i*, *j*) and (*by*(*i*, *j*) may be negative since there exists subtraction operation in step 2) of City-Block-Distance, so *bx*(*i*, *j*), *r*_1_^*i*^, *by*(*i*, *j*),and *r*_2_^*i*^ all need an additive bit as the signed bits. Therefore, their bits lengths are set to be *ρ* + *δ* + 2 when they enter SPADIS. In Figure 5, the left two SUBs and a MUX serve to compute |*x*_*i*_ − *x*_*j*_′| and the right correspondences serve to compute |*y*_*i*_ − *y*_*j*_′|. The output of ADD is exactly the city block distance |*x*_*i*_ − *x*_*j*_′| + |*y*_*i*_ − *y*_*j*_′|. To reduce the complexity of whole circuit, we take the same method which has been used in SPADIS for Euclidean distance—the module does not output city block distance directly. Instead, it computes d*(*i*, *j*):

(11) d∗(i,j)={2τ′−1,if  |xi−xj′|+|yi−yj′|≥2τ′or  oij=1        τ′  low-order  bits  ofif  |xi−xj′|+|yi−yj′|<2τ′,   |xi−xj′|+|yi−yj′|,oij=0.

The bits length of d*(*i*, *j*) is *τ*′ which is that of the threshold *T*_*D*_. It is half of *τ* since the latter is the bits length of squared threshold. The gates of the subsequent circuit also employ this length, so the complexity of the circuit is remarkably reduced.  Table 4 gives the complexity for city block distance.

## 6. Experimental Results and Discussion

As described in the above sections, the shorter bits length for representing a minutia can significantly lead to lower communicational and computational overloads. However, the shorter bits length consequentially may decrease the accuracy of matching. Hence, in this section, we try to evaluate the performance of the matching system and the effect of the bits length on the accuracy through the experiments. To achieve the goals, we implemented the whole system and tested on FVC2002-DB1 fingerprint database [26]. The database contains 8 images of 100 fingers, thus 800 images in total. The size of each image is 388 × 374 pixels. Consequently, in order to represent each entry of a minutia completely, nine bits are required. The minutiae of each fingerprint were extracted and prealigned by using the algorithm of [27]. We considered the following three scenarios of the bits length: eight bits, seven bits, and six bits. That is, each entry of a minutia, *x*, *y*, and *θ*, was linearly mapped and rounded to 8-bit, 7-bit, and 6-bit integers, respectively. The genuine accept rate (GAR) and false accept rate (FAR) are tested in accordance with the requirements of FVC2002. For a genuine match, each impression of each finger was compared with other impressions of the same finger. There were totally 28 combinations per finger and a total of 28 × 100 = 2800 tests that were done for GAR. The cross tests were also done among 100 fingers to evaluate FAR. The first impression of each finger was compared with the first impression of other fingers. Totally, 99 × 100/2 = 4950 tests were done.

There are several implementation tools of a generic secure two-party computation that have been developed in the past few years which serve to build privacy-preserving protocols, for example, Fairplay [28], TASTY [29], and FSCUGC [30]. The approach of FSCUGC allows the users to write their programs with a combination of high-level and circuit-level Java code and provides more efficiency and scalability than others with the pipeline technique. So, we employed Java and the library provided by FSCUGC implemented the protocols. In our experimental setting, the server (Bob) and the client (Alice) were set up on different PCs connected by a LAN. Both PCs were configured with AMD 1055T 2.8 G CPU and 8 GB DDR3 memory. We used Paillier encryption scheme with a 1024-bit modulus and 80 bits for symmetric and statistical security. The other parameters of the protocols used in our experiments are listed in Table 5.

Table 6 gives the average running time of our protocols on FVC2002-DB1, in which “*Enc*” refers to the time related to Paillier encryption; “*OT*” refers to that of Oblivious Transfer; and “*Circuit*” refers to that of garbled circuit. “Prep,” the preparation phase, only needs to be performed once. Since garbled circuits consume a large amount of memory with the increment of *N*_*T*_ × *N*_*Q*_, and the average number of the minutiae of a fingerprint approximates to 43 in FVC2002DB1, in order to reduce an excessive exhaustion of the memory, the upper bounds of *N*_*T*_ and *N*_*Q*_ were both set to be 90. Accordingly, the excessive minutiae will be discarded. Step (1) of Euclidean-Distance or City-Block-Distance was computed in the preparation of “*Enc.*” In the preparation phase of “*Circuit,*” the gabled circuits for *N*_*T*_ = 90 and *N*_*Q*_ = 90 were generated, except for the wire labels and garbled tables which would be regenerated for each execution. The preparation phase of *OT* protocol depends only on the security parameters we choose. The “Exec” in Table 6 refers to execution phase which must be operated for each fingerprint. For the gabled circuits, since he had generated 90 × 90 circuits, in this phase, the server reset the corresponding wire labels according to actual *N*_*T*_ and *N*_*Q*_ and transferred them to the client. As expected, the average execution time of *Enc* in the case of city block distance is much smaller than that of Euclidean distance. However, the running time is dominated by the computation related to garbled circuits, so the average total time of city block distance is greater than that of Euclidean distance since the circuits of the former are more complex than those of the latter.

**Figure 6:**
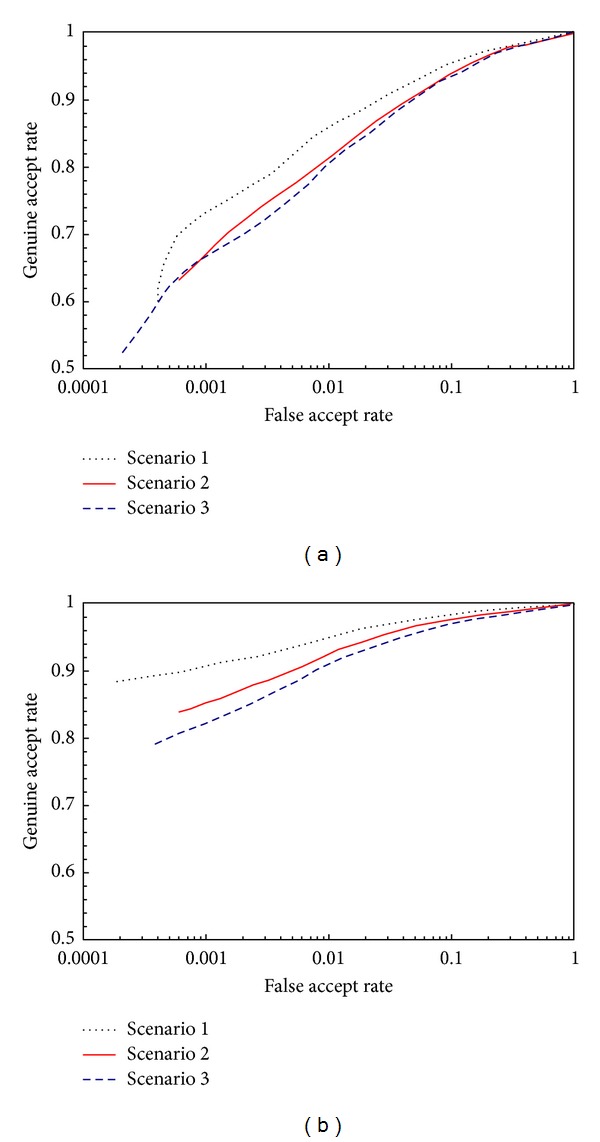
ROC curves for authentication. (a) Euclidean distance, (b) city block distance.

Table 7 shows the obtained accuracy measured by Equal Error Rate (EER), while Figure 6 shows the curves of receiver operating characteristic (ROC) of the matcher systems, in which Figure 6(a) is that of Euclidean distance and Figure 6(b) is that of city block distance. As expected, the quantization has effect on the accuracy of the matching algorithm. In both cases, the longer the bits length is, the higher the accuracy is. This can be explained by the fact that that quantization compresses the feature space. The two distinct minutiae with the distance less than a quantization step may be mapped to the same point. And the shorter the bits length is, the higher the probability is. This quantization effect increases FAR, so it lowers the accuracy of the matcher system. Surprisingly, the contrast between Figures 6(a) and 6(b) shows that the matcher based on city block distance has a better accuracy. The result can be explained as follows. We judge whether two minutiae are matched just according to the distance between them. The decision region formed by Euclidean distance is a circle whereas the one by city block distance is a diamond (square). For the same threshold *T*_*D*_, the radius of the circle is *T*_*D*_, while the side length of the diamond is $\sqrt{2}T_{D}$. Thus, the area of the diamond is only ${\left(\sqrt{2}T_{D}\right)}^{2}/\pi {\left(T_{D}\right)}^{2}\approx 63.7\%$ of that of the circle. As mentioned above, the quantization may lead to the merging of distinct minutiae. The bigger decision area, though it increases the GAR, meanwhile, increases FAR more. Hence, it reduces the overall accuracy of the matcher based on Euclidean distance. On the contrary, the smaller area lowers the probability of false matching, thus resulting in the improvement of the accuracy. Compared with the implementation of fingercode version of fingerprint [15], our schemes based on minutiae show a better accuracy performance when *T*_*D*_ = 15, *T*_*θ*_ = 20. And, compared with the results of [15], even six bits length also achieved a rather competitive accuracy (ERR = 0.051 for Euclidean distance and ERR = 0.045 for city block distance, resp.) when *T*_*D*_ = 15, *T*_*θ*_ = 20.

## 7. Conclusion

Biometry serves as an excellent mechanism for the authentication of individuals while biometric data are extremely sensitive and must be well protected. Furthermore, once leaked, the data cannot be revoked or replaced. The use of privacy-preserving protocol is a very desirable solution to improving security in biometric applications. In this paper we have addressed the construction of privacy-preserving protocols of fingerprint minutiae based on the combination of garbled circuit and homomorphic encryption. The proposed scheme provides protection for both template and transaction privacy. The template stored on the server is encrypted by the user's private key. Therefore, the template can be updated or revoked by reencryption. Two hybrid protocols with the combination of homomorphic encryption and garbled circuit are presented to fulfill the minutiae matching, in one of which, Euclidean distance is utilized as distance measures and in the other, city block distance is adopted. We have designed the efficient circuits to implement the corresponding tasks. The experimental results on FVC2002-DB2 show that the proposed scheme has acceptable verification accuracy. Future work could be oriented to the application of the results we obtained and to the development of privacy-preserving systems with a higher accuracy and efficiency.

## Figures and Tables

**Algorithm 1:**
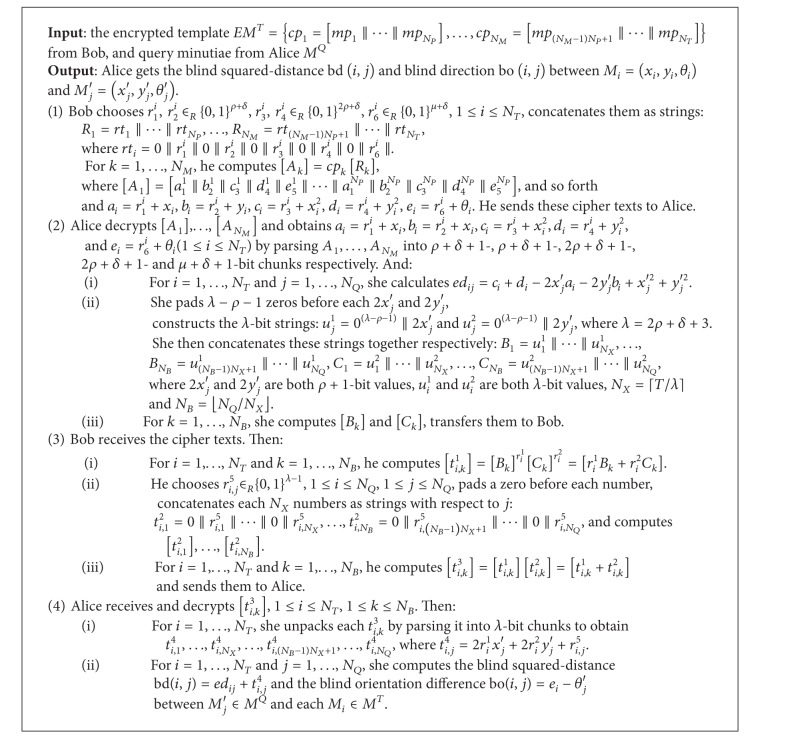
Euclidean-distance (*EM* ^*T*^, *M* ^*Q*^).

**Algorithm 2:**
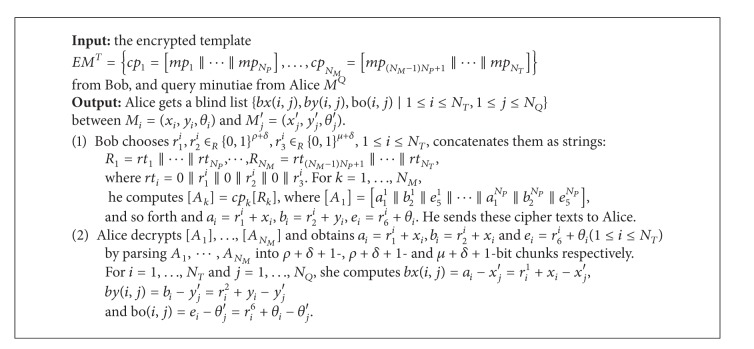
City-block-distance (*EM* ^*T*^, *M* ^*Q*^).

**Table 1:** Complexity of the protocol Euclidean-distance.

Round complexity	Communication complexity (bits)	Asymptotic computation complexity
3	Bob → Alice: (*N*_*M*_ + *N*_*T*_ × *N*_*B*_) × 2*T*	Bob: (*N*_*M*_ + *N*_*T*_ × *N*_*B*_)Enc + (2 × *N*_*T*_ × *N*_*B*_)Exp
	Alice → Bob: 2*N*_*B*_ × 2*T*	Alice: 2*N*_*B*_Enc + (*N*_*M*_ + 2 × *N*_*T*_ × *N*_*B*_)Dec

**Table 2:** Complexity of protocol City block distance.

Round complexity	Communication complexity (bits)	Asymptotic computation complexity
1	Bob → Alice : *N*_*M*_ × 2*T*	Bob: *N*_*M*_ (Enc)
		Alice: *N*_*M*_ (Dec)

**Table 3:** Number of non-XOR gates in circuit for Euclidean distance.

ORIDIFF	SPADIS	MIN2	MINM	COUNTER	MATCH	Total
77*μ* + 4*δ* + *σ* + 5	4*ρ* + 2*δ* + 12	2*τ*	2*τN*_*Q*_	m*	(4*ρ* + 2*δ* + 6 + 2*τ*)*N*_*T*_ *N*_*Q*_ +*τN*_*T*_ + *m*	(7*μ* + 4*ρ* + 6*δ* + *σ* + 17 + 2*τ*)*N*_*T*_ *N*_*Q*_ +*τN*_*T*_ + *m*

Note: *m = ⌈*N*_*T*_/2⌉ + 2⌈⌊*N*_*T*_/2⌋/2⌉ + ⋯+⌊log⁡*N*_*T*_⌋.

**Table 4:** Number of non-XOR gates in circuit for city block distance.

ORIDIFF	SPADIS	MIN2	MINM	COUNTER	MATCH	Total
77*μ* + 4*δ* + *σ* + 5	8*ρ* + 8*δ* + 10	2*τ*′	2*τ*′*N*_*Q*_	m*	(8*ρ* + 8*δ* + 10 + 2*τ*′) × *N*_*T*_ *N*_*Q*_ + *τ*′*N*_*T*_ + *m*	(7*μ* + 8*ρ* + 12*δ* + *σ* + 15 + 2*τ*′) *N*_*T*_ *N*_*Q*_ + *τ*′*N*_*T*_ + *m*

Note: *m = ⌈*N*_*T*_/2⌉ + 2⌈⌊*N*_*T*_/2⌋/2⌉ + …+⌊log⁡*N*_*T*_⌋.

**Table 5:** Bits length of parameters in our experiments.

	*ρ*	*μ*	*δ*	*σ*	*τ*/*τ*′*τ*/*τ*′	*η*_*r*_1__	*η*_*r*_2__	*η*_*r*_3__	*η*_*r*_4__	*η*_*r*_5__	*η*_*r*_6__	*λ*
Scenario 1	8	8	8	4	8/4	14	14	22	22	24	14	25
Scenario 2	7	7	7	3	6/3	13	13	20	20	22	14	23
Scenario 3	6	6	6	2	4/2	12	12	18	18	20	14	21

**Table 6:** Running time of the proposed methods.

		Euclidean distance			City block distance		
Average times		Scenario 1	Scenario 2	Scenario 3	Scenario 1	Scenario 2	Scenario 3
		s	s	s	s	s	s
	*En* *c*	0.158	0.158	0.158	0.048	0.033	0.031
Prep	*OT*	0.703	0.703	0.696	0.703	0.703	0.698
	*Ci* *rc* *ui* *t*	20. 818	16.392	14.010	28.974	25.869	22.893
	*En* *c*	3.301	3.089	2.991	0.048	0.030	0.028
Exec	*OT*	3.980	3.465	2.904	5.291	4.308	3.497
	*Ci* *rc* *ui* *t*	9.732	8.870	8.684	15.475	13.238	11.772

Average total times		17.013	15.424	14.579	20.814	17.576	15.297

**Table 7:** Accuracy performance of the proposed methods.

ERR	Euclidean distance			City block distance		
	Scenario 1	Scenario 2	Scenario 3	Scenario 1	Scenario 2	Scenario 3
T_D_T_D_ = 15, T_θ_ = 20	0.039	0.05	0.051	0.025	0.036	0.045
T_D_ = 20, T_θ_ = 30	0.087	0.092	0.095	0.06	0.075	0.082
